# Representation Learning and Pattern Recognition in Cognitive Biometrics: A Survey

**DOI:** 10.3390/s22145111

**Published:** 2022-07-07

**Authors:** Min Wang, Xuefei Yin, Yanming Zhu, Jiankun Hu

**Affiliations:** 1 School of Engineering and Information Technology, University of New South Wales, Canberra, ACT 2612, Australia; maggie.wang1@adfa.edu.au (M.W.); xuefei.yin@unsw.edu.au (X.Y.); 2 School of Computer Science and Engineering, University of New South Wales, Sydney, NSW 2052, Australia; yanming.zhu@unsw.edu.au

**Keywords:** biometrics, biological signal, classification, deep learning, feature extraction, pattern recognition, representation learning

## Abstract

Cognitive biometrics is an emerging branch of biometric technology. Recent research has demonstrated great potential for using cognitive biometrics in versatile applications, including biometric recognition and cognitive and emotional state recognition. There is a major need to summarize the latest developments in this field. Existing surveys have mainly focused on a small subset of cognitive biometric modalities, such as EEG and ECG. This article provides a comprehensive review of cognitive biometrics, covering all the major biosignal modalities and applications. A taxonomy is designed to structure the corresponding knowledge and guide the survey from signal acquisition and pre-processing to representation learning and pattern recognition. We provide a unified view of the methodological advances in these four aspects across various biosignals and applications, facilitating interdisciplinary research and knowledge transfer across fields. Furthermore, this article discusses open research directions in cognitive biometrics and proposes future prospects for developing reliable and secure cognitive biometric systems.

## 1. Introduction

Cognitive biometrics is an emerging branch of biometric technology complementing traditional biometric modalities that relies on physiological characteristics (what we possess) and behavioral characteristics (how we behave) by further incorporating ‘the way we think, feel, and respond’. We define the scope of cognitive biometrics as those based on biosignals from the brain, heart, and autonomic nervous systems, since these biosignals carry information of cognitive and/or emotional processing, reflecting the cognitive and emotional characteristics of a person. Unique physiological traits in terms of anatomical structures [1,2], intrinsic behavioral traits in terms of the thinking manner and preferences [3], and cognitive and emotional characteristics [4] together form the basis of cognitive biometrics.

Cognitive biometrics based on biosignals offer advantages in terms of privacy compliance [5], robustness against circumvention, intrinsic liveness detection, and user protection. First, these biosignals are results of cerebral, cardiac, or nervous system activity. They are internal traits that are not exposed to the public and many features are non-volitional, which means that the user cannot deliberately divulge their identifiers [4]. Moreover, with current sensing technology, it is unlikely that these biosignals could be captured covertly or remotely without the user’s conscious engagement, making cognitive biometrics less prone to spoofing attacks [6]. In addition, cognitive biometrics inherently support liveness detection and continuous applications, which protects the users and reduces the possibility of presentation attacks using spoof artifacts or lifeless bodyparts. Furthermore, cognitive biometrics is potentially cancellable, since biosignals are not static. For example, brain biometrics based on event-related potentials allow replacing a compromised biometric identifier with a new one that is elicited by a different stimulus or event [7]. This feature is not possessed by traditional biometric modalities such as fingerprint, palmprint, and iris. These advantages pave the way for new cognitive biometrics-based applications and have inspired a large body of publications in recent years. It is necessary to review the latest research results and point out future development directions.

To clarify our motivations and differentiate our survey from others, we provide a summary of the related works. Campisi et al. [1] investigated the characteristics and neurophysiological evidence of biometrics based on brain waves (i.e., EEG) and reviewed the elicitation protocols, acquisition, and recognition methods for EEG-based biometric systems. Gui et al. [7] continued the survey on brain biometrics, extending the review to the acquisition, collection, processing, feature extraction, and classification of EEG-based biometric systems. Similar surveys were also conducted for specific application scenarios, including EEG-based user verification [8] and EEG-based subject identification [9]. Rathore et al. [2] reviewed heart biometrics and discussed the challenges in various cardiac domains and prospectives for developing heart biometric systems. These surveys have mainly focused on a small subset of cognitive biometric modalities without considering the similarities and connections among the modalities. Blasco et al. [10] reviewed biometrics based on a user’s physiological and behavioral traits collected with wearable devices. Maiorana [11] continued the survey on wearable devices-based biometrics with a categorization of biometric traits. This is an overview of the maturity of wearable biometrics research, not a comprehensive review of the recognition systems. Cognitive biometrics overlaps with wearable device-based biometrics from the perspective of data acquisition, since some biosignals for cognitive biometrics can be captured by wearable devices, such as ECG and PPG. However, they are different topics.

**Figure 1:**
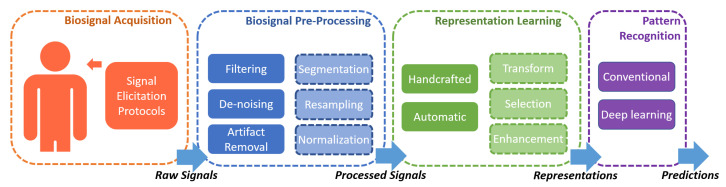
A typical cognitive biometric recognition system.

In summary, cognitive biometrics is attracting increasing attention from both academics and industry, but there is so far no comprehensive survey on cognitive biometrics that breaks the barriers among different biosignals and application scenarios. Figure 1 illustrates the typical structure of a cognitive biometric recognition system. This survey covers each component. Meanwhile, recent years have witnessed the progress of advanced machine learning models for processing biological signals in cognitive biometric recognition systems. The latest research output, especially the generative models for learning representations and various deep learning models for pattern recognition across different biosignals and applications, should be systematically reviewed. This study is a comprehensive review of cognitive biometrics to facilitate interdisciplinary research and knowledge transfer across fields, with specific contributions as follows:

- A comprehensive review on cognitive biometrics is presented, which covers all the major biosignal modalities and applications;
- A taxonomy is designed to structure the corresponding knowledge of cognitive biometrics and guide the survey;
- We provide a unified view of the methodological advances in signal acquisition, pre-processing, representation learning, and pattern recognition over various biosignals and applications. In particular, the latest developments of generative learning models and deep learning models in cognitive biometric recognition are included;
- A summary of the representative publicly available databases for cognitive biometrics is presented.
- We identify open issues and suggest future research directions for cognitive biometrics in machine learning, security, fusion, and persistence.

The remaining contents are arranged as follows. Section 2 proposes a taxonomy of cognitive biometrics and summarizes the application scenarios. Section 3 reviews the biosignals for cognitive biometrics and their acquisition and preprocessing approaches. A list of publicly available databases for cognitive biometric research is also summarized. Section 4 and Section 5 review the representation learning and pattern recognition in cognitive biometrics, respectively. Section 6 summarizes the open research directions, followed by a conclusion in Section 7.

## 2. Taxonomy of Cognitive Biometrics and the Applications

**Figure 2:**
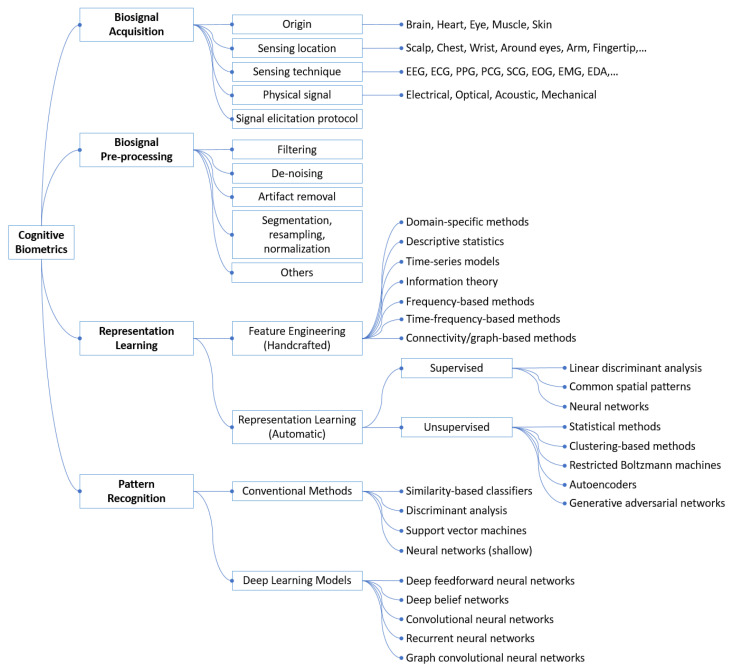
A taxonomy of cognitive biometrics.

In this section, we propose a taxonomy for cognitive biometrics, which consists of four aspects: biosignal acquisition, biosignal pre-processing, representation learning, and pattern recognition, as illustrated in Figure 2. The following sections will review each aspect with an emphasis on representation learning and pattern recognition.

**Figure 3:**
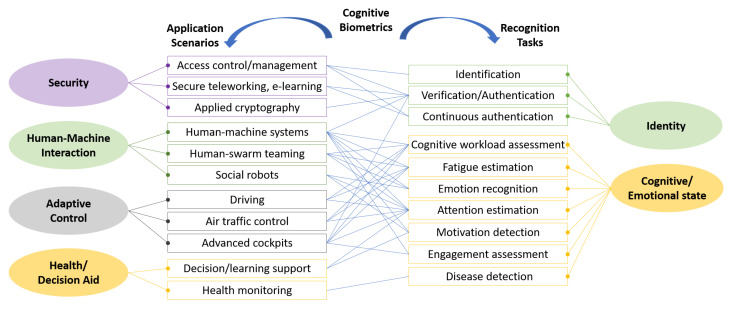
Application scenarios of cognitive biometrics and recognition tasks.

Due to the nature of cognitive biometrics, the application of cognitive biometrics is not limited to personal identification and verification, but also includes a wide range of scenarios from human-computer interaction to adaptive control and decision support. Figure 3 depicts the application scenarios of cognitive biometrics, the corresponding recognition tasks, and the connections between them.

The application scenarios are summarized into the following groups:

- Security management. This is the main function of cognitive biometrics, and the application scenarios include user login, access control, secure telecommuting and e-learning, and biocryptographic systems [12];
- Human–computer interaction. Effective human–machine interaction requires that the machine to adapt its behavior according to the user’s cognitive and emotional states, behaviors, performance, and other personal information including identity [13]. Cognitive biometrics support dynamic recognition of a user’s identity and cognitive and emotional states [14] and, therefore, are important tools for human–machine interaction in social robots, human–machine systems, and human–swarm teaming systems [15];
- Adaptive control. In closed-loop human–machine adaptive systems, the adaptive control aims to automatically update system parameters by associating the user and system states, so that the user and system can work together effectively and harmoniously. Cognitive biometrics provides informative and dynamic human state indicators, which are the basis for the adaptive control module to make decisions. Such adaptive systems are used in automatic driving, air traffic control, advanced cockpits, and other augmented cognition systems where recognition of the user’s cognitive workload [16,17], attention [18], fatigue [19], and engagement states is achieved through biosignals of cognitive biometrics (e.g., EEG, ECG, PPG, EOG, and EMG) [20];
- Decision and health aids. Other application scenarios include decision support and education assistance. In such applications, cognitive biometrics are used to estimate the user’s motivation, emotional state, and attention level to support the decision-making process or improve the learning concentration [18]. Cognitive biometrics based on biosignals from the brain and heart also possess inherent functions of monitoring the brain and heart health conditions and diseases.

Although the application scenarios vary, the core function of the cognitive biometrics module can be summarized as recognizing a person’s identity or a person’s cognitive/emotional state.

- Person identification. This answers the question ‘who is the person?’ by solving a one-to-N comparison problem. A person identification system takes a person’s biometric data and compares it to a database of possible candidates in order to decide the identity of the person. The performance of person identification systems is evaluated with the correct recognition rate. In addition, when considering persons not included in the database, the false positive identification rate is used to measure how good a system is at identifying unregistered persons.
- Verification (authentication). This answers the question ‘are you who you claim you are?’ by solving a one-to-one comparison problem. An authentication system takes a user’s biometric data and claimed identity and verifies whether the user is who that person claims to be. Typical evaluation metrics for verification are false non-match rate (FNMR) and false match rate (FMR), which measure the error rate of a genuine user being falsely rejected as an impostor and the error rate of an impostor being falsely accepted as a genuine user, respectively. Furthermore, researchers often generate detection error trade-off (DET) or receiver operating characteristic (ROC) plots to visualize the change of FNMR and FMR at different thresholds, from which the equal error rate (EER) is obtained.
- Emotion recognition. Correct recognition of a user’s emotional state plays an important role in human–computer interaction. Since emotions are complex psycho-physiological processes that are associated with thoughts, feelings, and behavioral responses, it is natural to use cognitive biometric signals for emotion recognition. Existing studies usually classify emotional states into two classes (e.g., positive and negative), three classes (e.g., positive, neutral, and negative), or more (happy, sad, neutral, and fear) [14]. Audio and visual stimuli, such as movie clips, are often used to evoke emotions in the subjects, and the collected data are labeled according to the stimuli presented and the subjects’ self-assessments.
- Cognitive workload assessment. Cognitive workload describes the level of mental resources utilized by a person in performing a task. Depending on the tasks, there can be different levels, usually from three to five [16,17].
- Cognitive fatigue (vigilance) estimation. A human presents different vigilance states, such as awake, tired, and drowsy [19]. A vigilance estimation system aims to recognize the user’s vigilance state and to detect fatigue or drowsiness. Real-time vigilance estimation plays an important role in improving driving safety and facilitating effective human–machine teaming.
- Attention recognition. The attention state of a human can change while performing a task; an attention recognition system aims to detect the change and recognize the attention level (e.g., low, neutral, high) [18]. Other recognition tasks include motivation detection, engagement assessment, and disease detection.

From a computer scientist’s perspective, cognitive and emotional state recognition is essentially a feature extraction and classification problem. Therefore, the performance evaluation of such recognition systems uses typical classification evaluation metrics, including accuracy, precision, recall, F1 Score, etc. ANOVA analysis is also used in vigilance estimation. The class labels are usually obtained through self-reporting or are determined by the tasks. In addition, it is worth mentioning that there are three types of methods to assess cognitive states (workload, vigilance, and attention) of users, including subjective measures (which are in the form of questionnaires), performance measures (such as error rate, reaction speed, and task completion time), and physiological measures such as EEG/ERP, EOG, EMA, EDA, heart rate, and heart rate variability. The physiological measures provided by cognitive biometric signals are superior to the others as they are objective, implicit, continuous, and able to be integrated into the system in an unobtrusive manner.

In different applications of cognitive biometrics, although the targets are different, the recognition systems share a similar structure as presented in the taxonomy in Figure 2. This taxonomy integrates knowledge and technologies from different domains, signals, and application scenarios for cognitive biometrics.

## 3. Biosignal Acquisition and Pre-Processing for Cognitive Biometrics

### 3.1. Biosignal Acquisition

To provide insights into how the acquisition and characteristics of different biosignals are related, we categorize biosignals of cognitive biometrics considering the following five dimensions: (1) sensing technique, which is related to the sensor and signal acquisition device; (2) origin, this is, the original body part that generates the biosignal; (3) sensing location, which describes where the sensors are placed on the body; (4) physical signal, which indicates the kind of physical signal that the sensor reads; and (5) elicitation protocol, indicating whether the recorded signal is a result of a spontaneous activity or evoked by internal tasks or external stimulation. Table 1 lists biosignals of cognitive biometrics in existing studies based on these five dimensions. We only consider sensing technologies that are lightweight, non-invasive, and suitable for practical application scenarios.

#### 3.1.1. Brain Signals

Brain signals used in cognitive biometrics are measured by electroencephalograph (EEG). EEG captures the electrical activity of the cortex from the scalp by placing electrodes at specific locations defined by relevant standards, for example, the international 10–20 system. For biometric applications, EEG signals are collected while the user is engaged in a particular task, where the task impacts the nature of the signal elicited. We classify the signal elicitation protocols used in EEG biometrics into resting-state protocols, internal stimulation protocols, and external stimulation protocols. The resting-state protocol requires users to rest with their eyes open or closed [21,22]. The recorded signal is continuous and reflects the spontaneous activity of the brain. The internal stimulation protocol elicits the desired brain responses associated with higher cognitive processing using internal or volitional tasks, such as pass-thoughts, motor imagery, imagining singing, math calculation, and other mental tasks [3]. External stimulation protocols, on the other hand, utilize external sensory stimuli, including visual, auditory, and somatosensory stimuli [23,24,25,26], to evoke activity in particular brain functional areas. In internal and external stimulation protocols, users usually need to repeat the task multiple times or receive repetitive sensory stimulation. Then EEG signals recorded in multiple trials are segmented and averaged to generate an event-related potential (ERP), which reflects the brain activity elicited by the adopted mental task or stimulus. This type of method is often referred to as ERP biometrics [4,27]. The resting-state protocol provides continuous data and ease of deployment, while the internal and external stimulation protocols provide higher signal-to-noise ratios and intra-subject stability, as the user’s mental state is under experimental control.

#### 3.1.2. Heart Signals

Heart activity can be measured from the chest and finger using different sensing techniques, including electrocardiography (ECG), photoplethysmography (PPG), phonocardiography (PCG), and seismocardiography (SCG). Specifically, ECG captures the electrical activity of the heart using electrodes placed on specific locations along the chest area. The primary features of ECG signals are the P wave, QRS complex, and T wave. Extraction of these features requires localizing the fiducial points of ECG signals. PPG measures the reflected light from the fingertip, earlobe, or forehead using a pulse oximeter. The signal reflects variations in the volumetric blood flow in the peripheral circulation, in which the alternating component provides heartbeat information. The collected data is a timeseries composed of periodic waves. PCG captures the acoustic signals generated by the heart during cardiac activity using integrated microphones. The collected data is a timeseries that contains two components, S1 and S2, in each cardiac cycle. These two components correspond to the closure of the atrioventricular valve and the semilunar valve, respectively, and they exhibit different frequency domain characteristics and duration. SCG records the mechanical vibrations induced by cardiac activity and heartbeat from the chest using accelerometers. SCG provides an inexpensive and convenient method to acquire cardiac activity, but its signal quality is lower compared to ECG due to exposure to ambient noise and motion artifacts during data collection.

#### 3.1.3. Muscle, Skin, Eye-Related Signals

Electromyography (EMG) measures the electrical activity produced by skeletal muscles. EMG signals reflect the user’s muscle activation level and movement, and are often used for gesture recognition, device control, and as an indicator to detect muscle fatigue in addition to biometric applications. Electrodermal activity (EDA) reflects skin conductance, which varies with the sweating condition of the skin. EDA contains two components, in which the phasic component in response to a distinct stimulus is called an event-related electrodermal response, and EDRs without an observable external stimulus are referred to as nonspecific EDRs. EDA offers a convenient way to detect changes in autonomic nervous system activity, which is associated with the user’s emotional and cognitive states. Electrooculography (EOG) captures the potential changes induced in eye movements between two electrodes attached around the eye, either horizontally or vertically. The collected data is a timeseries in which the direction of eye movement can be identified from different patterns in the signal. In addition to EOG, eye-tracking glasses and remote eye-trackers are also used to assess users’ behaviors and cognitive states in human–computer interface systems [28], especially for driver attention detection. The pupil response collected through eye-tracking devices is associated with the user’s concentration or emotional state [29]. EMG, EDA, EOG, and other eye movement signals are often used as auxiliary modules of multi-modal biometric systems to improve overall performance and robustness [14,30].

### 3.2. Publicly Available Databases and Biosignal Pre-Processing

The representative databases of the above-mentioned physiological signals for cognitive biometrics are summarized in Table 2. Although in many studies researchers have collected private data to evaluate their developed methods, the publicly available databases offer a better platform to compare different methods. These representative databases are selected since they provide data with a sufficient sample size from a relatively large number of subjects in well-controlled protocols or conditions. Some of them provide recordings of multiple sessions, an important condition for cross-session evaluation.

**Figure 4:**
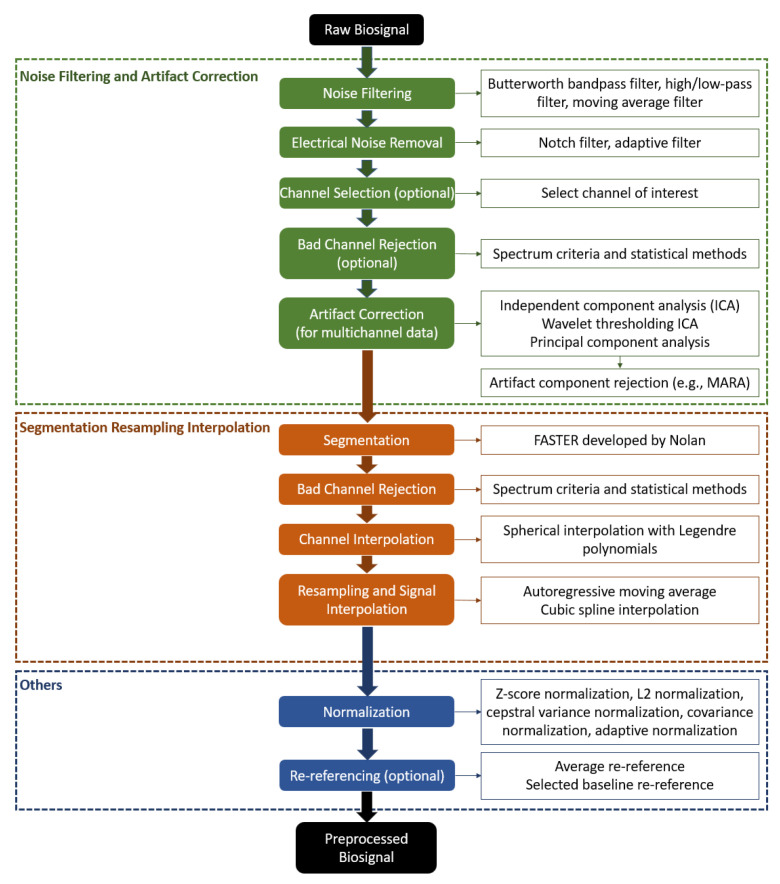
Biosignal pre-processing for cognitive biometrics.

The acquisition of different cognitive biosignals employs sensors and devices with different hardware configurations, sensing locations, sampling rates, and other specifications. Signal pre-processing is a necessary step to reduce noise and artifacts accumulated during data collection in order to better extract true biometric features from the collected biosignals. We summarize a unified pre-processing framework for major biosignals used for cognitive biometrics (such as EEG and ECG), as in Figure 4. However, it is worth mentioning that the specifics of signal pre-processing depend on the type of the signal. For example, the pre-processing of EDA involves specific steps, including separating the tonic and phasic components, identifying the onset and offset points, and estimating the trend of the phase component [31]. In addition, the choice of pre-processing technical details depends on the signal acquisition equipment, configuration, and the actual application scenario. Selecting appropriate pre-processing techniques requires taking into account the signal acquisition equipment, configurations, data acquisition protocol and condition, and application scenarios. In the following study of this survey, we will look into the details of the sensors, devices, acquisition configurations, and pre-processing techniques for cognitive biometric systems.

For multimodal approaches, synchronization between events/stimuli and the evolution of biometric signals is a critical issue. Synchronization is essential because EEG, ECG, or EDA acquisition devices may operate at different sampling rates, and these different data streams should be aligned in time. Event-based synchronization [32,33] is often adopted in multi-model cognitive biometric systems, where each data stream is time-stamped and aligned with a local clock or stream sequence number. Available tools for time-synchronization and networking include the lab streaming layer [34], which is a system designed for the unified collection of multiple data streams from different sensors and devices, and between programs and computers.

## 4. Representation Learning in Cognitive Biometrics

Feature extraction, or representation learning, is a critical step in recognition systems. It constructs informative and discriminative derived representations from a set of biosignal data to facilitate the subsequent classification and generalization steps and provide better interpretations. The quality, robustness, and generalizability of the representations directly affect the recognition performance and reliability of the systems. This section examines representations used in cognitive biometric recognition systems.

### 4.1. Handcrafted Representations

Handcrafted representations refer to features manually engineered based on domain knowledge of the signal. Considering different characteristics of signals, handcrafted features can be extracted by the following six groups of methods.

#### 4.1.1. Domain-Specific Methods

This type of method relies on domain knowledge of the specific signals. Typical representations include those based on fiducial points of the signals; particular peaks, intervals, and other morphological characteristics related to fiducial points; peaks, and intervals. They reflect the time-domain information of the signal in terms of shape, amplitude, and morphological structure. For cardiac signals (ECG, PPG, PCG, and SCG), the fiducial points are detected to segment a normal rhythm into multiple entities (e.g., P wave, QRS complex, and T wave for ECG signals), from which morphological features relative to the peak, interval, amplitude, slope, angle, and power ratio are extracted [35,36,37,38,39]. Domain-specific representations for EOG signals include the energy features that describe the signal amplitude, peak position features, slope features that characterizes the degree of sharpness, and the derivative features that represent energy and position features for the first derivative signals [40]. For the brain signals, domain-specific representations are usually used for ERP and VEP signals and contain particular peaks and valleys in response to certain sensory stimuli [24] and specific waveforms that are time-locked to elaborately designed events [4,27]. The extraction of domain-specific features relies on peak/fiducial point detection and wave segmentation algorithms. For example, the Pan–Tompkins algorithm is widely used for real-time location of QRS complexes from ECG signals [41]. It uses a series of filters for noise signals, emphasizes the QRS contribution by a derivative filter and squaring, and finally applies adaptive thresholds to detect the peaks of the filtered signal. Other peak detection methods are proposed based on the Shannon energy envelope, Hilbert-transform (HT), and moving average (MA) filtering [42]. For EEG signals, the segmentation of event-related potentials and peaks also relies on the human inspection and the signal acquisition protocols [4,24].

#### 4.1.2. Descriptive Statistics

In cognitive biometrics, descriptive statistics are generally applicable to extract features from all signals and are not limited to specific fields. The most widely used statistical representations can be divided into three categories, which describe the central tendency (mean, median, and mode) [43,44,45], dispersion (variance, standard deviation, range, and quartiles) [43,45,46], and shape (skewness and kurtosis) of the data [43], respectively. Other statistical representations include the maximum and minimum value of a segment, and the number and rate of zero-crossings (a zero-crossing is a point where the signal waveform intercepts the zero or mean value) [43,47]. In these works, descriptive statistical features are either extracted from the signal timeseries or calculated on top of other feature vectors (such as power spectral density features).

#### 4.1.3. Time-Series Models

An autoregressive (AR) model describes the time-varying processes in signals by specifying that the value of the timeseries at a certain time depends linearly on its own previous values and on a stochastic term (white noise). AR coefficients are popular EEG features that capture time-dependency information in the signal [21,48,49,50,51,52]. There are two main ways for fitting an AR model to an EEG signal, namely the Yule–Walker method and the Burg method. The Yule–Walker method solves the problem using the auto-correlation function, which is estimated by the covariance function of the signal. It requires calculating matrix inversion and the whole computation process needs to be repeated with a different order *p*. The Burg method recursively computes the parameters of AR(*p*) using parameters of AR(p−1) based on the Levinson recursion while estimating the parameters by minimizing the forward and backward prediction errors at the same time. It is more efficient and therefore more popular for feature extraction in EEG [53]. For the Berg method, the reflection coefficients are also used as features in addition to the AR coefficients [53,54,55,56]. Extension of the AR model includes the multivariate AR (mAR) model, where the value of each variable at a certain time is predicted from the historical values of the same series and those of all other timeseries. Parameters of the mAR model are potentially more informative features for EEG than the AR parameters, as they incorporate physiologically relevant connections between signals from different channels [57].

#### 4.1.4. Information Theory and Complexity

Entropy, a key measure in information theory, quantifies the amount of uncertainty involved in the value of a random variable. It is an effective tool to evaluate the dynamic complexity of biological signals, especially for EEG, ECG, and EDA. For different subjects, their signals may contain complex dynamics at different levels; therefore, transforming the information encoded in the dynamics into appropriate entropy features with discriminatory powers is one way to achieve cognitive biometrics. Entropy is also an effective biomarker that has been widely used in clinical applications such as seizure detection in epileptic patients [58]. The entropy estimation methods widely used in cognitive biometrics are approximate entropy (ApEn) [59], sample entropy (SampEn) [60,61], and fuzzy entropy (FuzzEn) [62]. ApEn describes the unpredictability or randomness of a finite length signal by embedding the signal into a phase space and estimating the increment rate of patterns within a predefined value when the embedding dimension is increased by one. SampEn improves ApEn by being less dependent on the length of the timeseries. FuzzEn extends the hard and precise similarity boundary in previous entropy measures into a smooth and contiguous boundary by replacing the Heaviside function with a fuzzy membership function (e.g., the family of exponential function) [63,64]. This extension makes FuzzEn a better fit for biological data, since the uncertainty at the boundaries between classes can provide a shade of ambiguity. Existing findings also suggest that FuzzEn is potentially a more reliable entropy measure for physiological data compared to non-fuzzy measures, especially when the signal is short in length and contaminated with noise [58]. In addition, differential entropy, which extends the idea of Shannon entropy, has been demonstrated to be an effective feature for EEG emotion recognition [14,65]. Moreover, in some studies, entropy is estimated after decomposing signals into multiple frequency bands or mode functions (e.g., empirical mode decomposition) to gain finer representations [58,60,64]. Other complexity measures such as correlation dimension and Lyapunov exponents are proposed for ECG signals [66].

#### 4.1.5. Frequency-Based Methods

Power spectral density (PSD) reveals the power distribution of signals in the frequency domain and is one of the most popular methods for feature extraction in cognitive biometrics. To estimate the power spectrum, two types of approaches are proposed, namely, non-parametric approaches based on discrete Fourier transform (DFT) and fast Fourier transform (FFT) [22,59,67], and parametric approaches based on AR modeling using the Yule–Walker or Burg method [68]. Typical PSD-based representations for EEG include raw PSD values [22,59,67] and their derivatives such as the Welch’s averaged modified periodogram [48,53,69,70], the spectral distribution [71], variance of spectral power, region of the power spectrum [59,72], spectral power and energy of selected frequency bands [48,73,74], and power ratio of selected bands [75]. The selection of frequency bands is often achieved by power spectrum analysis or digital bandpass filters (e.g., Butterworth filter) [48,73]. The short-time Fourier transform (STFT) extends the Fourier transform to reveal how the frequency content of physiological signals changes over time, thereby incorporating non-stationary characteristics. A Hamming window is usually used to divide the signal into segments where DFT is computed on each segment. The resulting STFT coefficients are important time-frequency representations for EEG, ECG, SCG, PCG, and EDA signals [76,77,78,79,80]. Continuous wavelet transform (CWT) also captures temporal and spectral information simultaneously [81]. It operates convolution on the input data timeseries with a set of functions generated by the mother wavelet. Compared to STFT, CWT offers variable time-frequency resolution preserving time shifts and scaling, and provides a flexible choice of the wavelet functions, among which the Morlet/Gabor wavelet is preferred for being closely related to human perception [82]. Discrete wavelet transform (DWT) with Haar wavelets and Daubechies wavelets are popular for EEG and ECG signals [59,83,84,85,86]. DWT differs from CWT in how the scale parameter is discretized. It provides sparse representations for cognitive biometric signals without high redundancy, as in CWT and STFT. Therefore, the coefficients of DWT are usually directly extracted into a feature vector for classification with conventional classifiers such as support vector machines, hidden Markov models, and discriminant analysis [83,87]. In contrast, STFT and CWT can be used to create two-dimensional time–frequency representations (often referred to as spectrograms and scalograms, respectively), which are then fed into convolution neural networks [76,77,88,89]. Wavelet packet decomposition (WPD) generalizes the DWT in preserving the detail coefficients, which capture the information lost between two successive approximation coefficients in each filtering step. Therefore, WPD offers a finer and potentially more robust representation than DWT [84]. The decomposition level varies in different studies; however, it is often set in the range of 3 to 5 to leverage between time and frequency domain information [17,84,86]. This is because the coefficients of a higher decomposition level reflect more frequency details; however, they retain less temporal information. Besides coefficients of the sub-bands, statistics and entropy features are extracted on top of the power or energy of the sub-bands [59,82,85,86,90]. In addition, coefficients of discrete cosine transform (DCT), autocorrelation-DCT, and Mel-frequency cepstrum (MFC) also demonstrate strong potential in providing multiple frequency-based features for EEG and ECG signals [35,36,55]. Existing studies also apply empirical mode decomposition (EMD) to decompose a signal into intrinsic mode functions [91] and use Hilbert–Huang transform (HHT) to obtain instantaneous frequency as features [92].

#### 4.1.6. Connectivity-Based Methods

Connectivity is defined to measure the relationship between biosignals of various regions/channels and has been widely used in the representation of EEG. In these studies, connectivity is calculated on multivariate signals using different statistical and effective metrics, as summarized in Table 3, where each metric measures the interaction from a particular perspective and defines a peculiar subjective connectivity network. Some studies directly concatenate these raw connectivity values into a feature vector for biometric recognition [21,22,25,93], while others extract node centrality features (such as degree and eigenvector centrality) from connectivity graphs [94,95]. A recent study [96] examines the impact of connectivity metrics and graph features (six nodal features and six global features) on EEG biometric identification. This analysis suggests that phase synchronization would bring more robust connectivity estimation than time-domain connectivity metrics, and a proper combination of connectivity metrics and features is necessary to achieve good identification accuracy and inter-state stability. In addition, deep learning models such as convolution neural networks and graph convolution neural networks are proposed to learn intrinsic structural representations from the EEG connectivity graphs [97,98]. Notably, an important aspect of connectivity-based methods is thresholding the functional connectivity matrices to reduce spurious connections, thereby restoring meaningful topological properties of the graphs [99].

### 4.2. Automatic Representations

Representation learning allows the automatic extraction of the representations needed for a recognition task, which offers a different perspective to feature engineering with explicit algorithms. It can be achieved in either a supervised or an unsupervised manner, as detailed below.

#### 4.2.1. Automatic Representations via Supervised Learning

(1.1) Linear Discriminant Analysis (LDA): Given a training set, the LDA finds a linear combination of features that separates different classes by maximizing the ratio of the inter-class to intra-class scatter matrices. The resultant combinations can be viewed as a new representation of the input data with a lower-dimension while preserving class separability. In cognitive biometrics, LDA is often used as a classifier, and more commonly, for feature transformation to reduce the dimensionality and improve separation before classification [68]. In these studies, LDA representations are obtained from a linear projection of the input signals and other features that are extracted by frequency-based methods (e.g., STFT in conjunction with DCT) [101] and PCA (e.g., the Eigenbrain projections) [72].

(1.2) Common Spatial Pattern (CSP): The CSP is a spatial filtering method that encodes the most discriminative spatial information of signals by maximizing the ratio of variance of two classes. Let $C_{1},C_{2}\in R^{N\times T}$ denote two classes of signals; the CSP filters F are obtained by

(1) $$F=\arg\max_{F}\frac{\|FC_{1}{\|}^{2}}{\|FC_{2}{\|}^{2}},$$

where the rows of F are the spatial filters. An original signal *s* can then be represented by projecting into the filter space by $\hat{s}=Fs$. CSP filtering is a popular method used in the cognitive biometrics domain for two-class classification tasks such as motor imagery classification and emotion recognition [102,103,104].

(1.3) Neural Networks: In cognitive biometrics, neural networks seek a mapping function between the input signals and class labels. They are a powerful tool for modeling biosignals, especially when the underlying relationship is complex and non-linear. In a multi-layer neural network, a representation of the input is learned at the hidden layers, which is subsequently used for classification at the output layer. In other words, the representations are data-driven and classification task-oriented, and therefore often achieve good classification results. Since the representations learned through neural networks are embedded in the classification task and are part of the classification model, we will discuss them in Section 5.

#### 4.2.2. Automatic Representations via Unsupervised Learning

(2.1) Statistical Methods: Principal component analysis (PCA) is widely used for pre-processing, feature extraction, and dimensionality reduction for EEG, ECG, EMG, and EOG signals [86]. Since EEG and cardiac signals often comprise a high degree of correlation between signals or features, PCA serves as a good tool to reduce signal or feature dimensions while retaining the most useful information [46,72,82,105]. Independent component analysis (ICA) is aimed at decomposing a multivariate source signal into multiple independent components by optimizing the statistical independence of the estimated components. ICA and its variants are widely used for removing artifacts from desired signals [106,107], and extracting and separating EEG, ECG, and EOG signals [108,109].

(2.2) Clustering-based Methods: Clustering is a process of dividing data into clusters such that each cluster holds the most similar data instances. Clustering algorithms used in cognitive biometric recognition include K-means [110,111,112], fuzzy K-means [113], and Gaussian mixture models (GMMs) [114]. K-means uses the data mean to update the center of each cluster and perform deterministic assignments. It is fast to compute, but is sensitive to outliers and only works well for certain data structures (e.g., convex data). GMMs generate multivariate Gaussian distributions and provide probabilistic assignments to clusters, therefore offering more flexibility than K-means. Fuzzy K-means extends K-means so that each instance has a fuzzy degree of belonging to each cluster. In the field of cognitive biometrics, clustering algorithms are used to obtain the inherent structure information in biosignals such as EEG and ECG [110], to detect waves from cardiac signals (e.g., detecting QRS-complexes from ECG) [111], and to pre-classify or transform extracted features to facilitate classification [112,114]. For example, Orhan et al. adopted K-means to cluster wavelet coefficient features for each frequency band and used the probability distributions as inputs to a neural network classifier [112]. In another study, a GMM was used as a fusion function to transform the multi-dimensional EEG representations extracted from an auto-encoder into a single representation that reflects the underlying statistical characteristics [114].

(2.3) Restricted Boltzmann Machines (RBMs): RBMs are a generative stochastic network architecture containing two layers: a visible layer and a hidden layer. The visible layer aims to represent the observable data, while the hidden layer captures the probability distribution of the features. In cognitive biometric recognition, RBMs have been successfully used for feature extraction and classification as a basic network block in deep belief networks [115,116,117]. In addition, RBMs are integrated with other neural network architecture for feature extraction and fusion. For example, RBMs are combined with autoencoders to learn and fuse representations from EEG and eye movement signals collectively for emotion recognition [14,115]. The results demonstrated that the extracted representations contain complementary information from internal cognitive states and external subconscious behaviors and hence enhance emotion recognition performance. Lu et al. [116] proposed a deep learning scheme based on RBM to generate a new representation of EEG features and achieve further performance improvement for motor imagery classification. Li et al. [117] proposed a multi-layer network ERP-NET based on the multi-channel temporal RBM to learn complex spatial and temporal patterns from single-trial ERP signals. They demonstrated that the ERP-NET is able to discover new ERP patterns and that the patterns learned by the ERP-NET are discriminative ERP components in which the ERP signals are properly characterized.

(2.4) Autoencoders (AE): AEs are an effective tool that learn data representation for cognitive biometric recognition from biosignals in an unsupervised manner. A basic AE structure consists of two neural network components, named the encoder and the decoder. The encoder maps data to feature space while the decoder produces a reconstruction of data by mapping the hidden code from feature space to data space. It is trained by minimizing a reconstruction loss function, $L\left(x,x^{\prime }\right)=||x-x^{\prime }{\left|\right|}^{2}$, where *x* and $x^{\prime }$ are the input and corresponding reconstruction by the autoencoder. Classical AEs are widely used for feature learning and dimension reduction (data compression) in ERP signal classification [118], EEG-based epilepsy detection [119], and emotion recognition tasks [120]. In these studies, other neural network constructs such as CNNs and LSTM are integrated into the AE architecture to gain finer representations that encode spatial and temporal information [118,119]. Moreover, Chai et al. [120] combined an AE network with a subspace alignment solution to constrain the distribution discrepancy, and their results demonstrated that this combination effectively improved emotion recognition accuracy. In addition, AEs with different training strategies (i.e., channel-wise or without differentiating the channel) have been analyzed for feature learning and visualization of short-time multi-channel EEG recording [121]. Autoencoders with a gate-control regularization are also proposed for EEG missing channel reconstruction [122].

Sparse AE and contractive AE are two variants that apply regularization constraints to the classical AE. Specifically, the sparse AE adds a non-linear sparsity term, $\alpha KL(\rho ||\hat{\rho }$, to encourage the sparsity of the learned representation, where $KL(\rho ||\hat{\rho }$ is the Kullback–Leibler divergence (relative entropy). The contractive AE penalizes the sensitivity of the representation with respect to the input using a regularization term, ${\beta ||J\left(x\right)||}_{F}^{2}$, which is based on the Frobenius norm of the Jacobian matrix of the encoder. A recent study combined CNN and sparse AE for EEG feature extraction in an emotion recognition task, in which the sparse AE was used to reduce redundancy from representations learned by the CNN [123]. Sparse AE provides a simple interpretation of the input data, while contractive AE makes the mapping from the input vector to the representation converge with higher probability [124]. Both of them improve the classification performance. Denoising AEs enhance the robustness of classic AEs to deal with noisy input by learning a denoising function [125]. The core idea is to add noises to the training data and force the AE to recover the noise-free version of the training data. Accordingly, the loss function for optimization in denoising AE is updated as $L_{D}\left(x,{\tilde{x}}^{\prime }\right)=||x-{\tilde{x}}^{\prime }{\left|\right|}^{2}$, where $\tilde{x}$ is the corrupted input of *x* by a preliminary stochastic mapping (Gaussian, salt and pepper, or masking) and ${\tilde{x}}^{\prime }$ is the reconstructed data. Denoising AEs exploit the statistical dependencies inherent in the input training data and eliminate the adverse effects of the noisy inputs corrupted in a stochastic manner. Existing findings suggested that denoising AEs are more effective than classical AEs in learning representations from biosignals, especially when these signals are contaminated with noise and artifacts [126]. Denoising AEs with sparse regularization are also proposed for EEG feature extraction to gain robust and sparse representations [127]. This method shows great potential in analyzing non-stationary epileptic EEG signals.

Variational AE introduces regularization of the latent space to encode the input as a distribution over the latent space instead of encoding it as a single point, as in previously discussed AEs. Since biosignals in cognitive biometrics usually follow certain statistical distributions, variational AE is more effective in learning matching representations that generate a distribution in the latent space with similar statistical properties to the input data [114]. A Gaussian distribution is often used in practical implementations, so that the loss function can be written as: $L_{V}\left(x,x^{\prime }\right)=L\left(x,x^{\prime }\right)+KL\left(N\left(\mu ,\sigma \right),N\left(0,1\right)\right)$, where the regularization term pushes the distribution generated by the latent representations to approach a standard normal distribution. Variational AEs have been widely used for learning representations from biosignals to enhance biometric recognition performance [114,128,129,130]. In these studies, the variational AEs were integrated with other constructs such as autoregressive layers [114] and graph neural networks [128] to capture dynamics from EEG timeseries for diverse classification tasks [114] and to learn graph embedding from EEG functional connectivity input [128]. Latent representations learned by variational AEs also show promising results in EEG-based emotion recognition [130] and ERP-based driver–vehicle interface systems [129].

(2.5) Generative Adversarial Networks (GANs): The GAN, proposed by Goodfellow et al. [131], is a framework to teach a deep learning model to learn the distribution of training data and thus to generate new data from that same distribution. A GAN is made of two neural networks, a generator and a discriminator. These two networks emulate a competition, where the generator takes a random vector sampled from a noise distribution as input and tries to generate samples as ‘real’ as possible, and the discriminator takes the generated samples as input and tries to distinguish them from the real samples. During training, the goal of the generator is to generate fakes to maximize the classification error of the discriminator while the goal of the discriminator is to beat the generator by identifying the generated samples. This zero-sum game is modeled as an optimization problem by:

(2) $$\underset{G}{min}\underset{D}{max}L\left(D,G\right),$$

where

(3) $$L\left(D,G\right)=E_{x∼p_{data}\left(x\right)}\left[log\left(D\left(x\right)\right)\right]-E_{x∼p_{z}\left(z\right)}\left[1-log\left(D\left(G\left(z\right)\right)\right)\right].$$

*D* and *G* are the generator and discriminator, respectively. *x* is the training data, $p_{data}\left(x\right)$ is the distribution of training data, and *z* is a noise following a distribution $p_{z}\left(z\right)$. Training of GAN is done in alternation between the discriminator and the generator to minimize the generator loss and maximize the discriminator loss. Independent back-propagation procedures are applied to both networks. GANs have the ability to learn any kind of data distribution in an unsupervised manner and have been successfully used in various domains such as computer vision, natural language processing, time-series synthesis, and semantic segmentation [132,133].

In the field of cognitive biometrics, GAN has been widely used for learning robust representation [134,135,136] and for data synthesis and dataset augmentation [81,137,138,139,140,141,142,143,144,145,146]. In these studies, the generator in the GAN framework was designed by different neural networks considering the characteristics of the input signals. For example, Abdelfattah et al. [142] adopted a recurrent neural network for the generator to learn the statistical characteristics of the time dependencies of input EEG signals. Zhang et al. [137] proposed a multi-generator conditional Wasserstein GAN method to generate a high-quality artificial EEG signal that covers a more comprehensive distribution of real data. The adoption of the multiple generators and the inclusion of label-based constraints enable the generators to learn various features and to learn the data patterns of real data from various perspectives. Panwar et al. [138] proposed a conditional Wasserstein GAN model with gradient penalty (cWGAN-GP) to synthesize EEG data for different cognitive events. By using a deconvolution layer with bilinear weights initialization and a two-step upsampling technique, this model addresses several EEG signal modeling challenges, including frequency artifacts and training instability. Hartmann et al. [139] proposed an EEG-GAN to generate naturalistic EEG signals by gradually relaxing the gradient constraint in WGAN-GP to increase the training stability. Similar cWGAN frameworks were also used to generate realistic EEG data in differential entropy form for data augmentation to enhance emotion recognition accuracy [140,141]. Moreover, cWGAN has been adopted in a driver sleepiness detection system to augment EOG data for training an LSTM classifier [81]. Zhu et al. [144] used a GAN composed of a bidirectional LSTM and CNN to generate synthetic ECG data. Debie et al. [134] proposed a privacy-preserving GAN model to generate and classify EEG data. It was trained under a differential privacy model to enhance the data privacy level by limiting queries of data from artificial trials that could identify the real participants from their EEG signals. Fu et al. [135] designed a conditional GAN to map EEG data associated with emotions to coarse facial expression images, and proposed a training strategy to achieve fine-grained estimation and visualization of EEG-based emotion. Caoley et al. [147] proposed a GAN-based deep EEG super-resolution approach to produce high spatial resolution EEG data from low-resolution samples. It can generate channel-wise up-sampled data to effectively interpolate numerous missing channels and thus increase the information contained in EEG signals. By adopting a modified sequence of convolutional layers, it allows EEG data to be processed across channels. Golany et al. [136] proposed a simGAN that incorporates the dynamics of ECG signals into the generative process for ECG classification. Specifically, it uses a system of ordinary differential equations to represent heart dynamics and incorporates this ODE system into the optimization process of the GAN to create biologically plausible ECG training examples. The ECG simulation knowledge learned by the simGAN has been proved to improve ECG classification. Shin et al. [146] proposed a GAN that uses ECG as input to generate photoplethysmogram (PPG). This study demonstrates that a GAN can be a potential tool to generate synthetic biosignals for data augmentation purposes in low-resource settings. Other generative adversarial models that could be useful for cognitive biometric data processing include the adversarial AE, which uses a GAN to perform variational inference for an AE.

### 4.3. Discussions

A summary and discussion of the handcrafted and automatic representations described in this section are as follows.

#### 4.3.1. The Handcrafted Representations

Domain-specific representations are intuitive, possessing physical meanings that can be interpreted by domain experts, and thus can be comprehended by a broad range of audiences. However, the extraction of these features requires precise localization of fiducial points or peaks in the signals and signal segmentation and alignment, which further increases the complexity. In addition, these features are sensitive to amplitude changes and noise, and their performance depends largely on good signal pre-processing.

Descriptive statistics-based representations reflect time-domain information in terms of the amplitude and shape of the signals from a statistical perspective. They are computationally efficient and universal to all signals, with no dependency on their domains. These representations can be affected by the length of signal segments; therefore, defining an optimal segment length is usually required to enhance performance. Another disadvantage is that they are susceptible to noise and amplitude fluctuations. For example, variations in the signal acquisition environment, equipment, and sensor placement, as well as human artifacts can easily introduce errors in the raw signals, which could lead to unstable representations or invalidate an established one.

Representations based on AR models capture the dependency relationship in signal timeseries using linear functions. The premise of using an AR model is that the input stochastic process is generalized as stationary. However, physiological signals usually present non-stationary characteristics and therefore do not always meet the premise. Therefore, a common practice is to segment signals with a moving window and assume stationarity within the short segment. AR parameters are important time-domain representations for EEG signals in many applications, including personal identification, authentication, and recognition of cognitive and emotional states. However, such representations have limitations, as they only reflect linear temporal dependency information of signal timeseries. A recent finding also suggests that AR representations are unstable and exhibit intra-individual variations that hinder the performance of biometric recognition systems [98].

Entropy is a useful explanatory tool for cognitive biometrics, because it provides a quantitative indicator of the randomness or complexity of dynamic signals, and at the same time describes its informational characteristics. However, as entropy is calculated on the signal timeseries, it inherits the shortcomings of being sensitive to amplitude fluctuations and noise. Moreover, it is a univariate feature that is extracted from single signals while ignoring the relationship between different channels. The discriminant power of entropy-based representations varies application-wise and depends largely on the recognition model.

Frequency-based methods have advantages in isolating noise and artifacts because the signals for cognitive biometrics have their valid frequency ranges. More importantly, for signals such as EEG, which are naturally described in terms of rhythmic activity in specific frequency bands, frequency-based representations reveal important information about the signal in these bands and offer links to interpret results with findings in neuroscience. Among the many methods, WT and WPD encapsulate temporal–spectral information simultaneously, thus providing richer representations than DFT and FFT. Compared with time-domain representations, frequency-based ones are more robust to intra-subject variability caused by cardiovascular conditions and cognitive/mental states. They are flexible to compute; however, they are less intuitive for human comprehension and usually rely on machine learning algorithms for classification. In cognitive biometric recognition, their performance is subject to a sufficient feature dimensional space and the recognition model.

Connectivity-based representations encode relational information between signals, which differs from univariate features that only capture characteristics of signals from individual channels. Being a bivariate measure, they are more robust against amplitude fluctuations of signals [22,98]. They are useful in reducing the intra-person and inter-session variations, a critical aspect in improving the performance of biometric recognition systems. However, the computation of connectivity requires multi-channel data collection. A sufficient number of channels is usually required to obtain robust representations, especially for graph-based methods [148].

#### 4.3.2. The Automatic Representation Learning Methods

LDA and CSP are two supervised learning algorithms for learning representations from cognitive biometric signals. The commonality of the two is that they both project data into a transformed space in which data of different classes are separable, while the difference is how they obtain the projection function. LDA can only separate data with different means, while CSP uses variance instead of mean, which makes CSP suitable for separating ERP from noise. CSP captures spatial information from multi-channel signals and is often used as a spatial filter for feature extraction. LDA, in contrast, is often used for feature transformation, which is a pre-classification procedure that aims to reduce dimensionality of extracted features while retaining discriminative information, or is directly used as a classifier.

Unsupervised representation learning serves different functions in cognitive biometric recognition systems. Specifically, PCA and ICA are useful tools for denoising and artifact removal and have been widely adopted in signal pre-processing. Moreover, they are able to transform feature vectors into a lower-dimension while preserving the most important information. Clustering methods (K-means and GMMs) are usually applied as a soft classification step to facilitate the final classification process. RBNs and AEs are mainly used for representation extraction, and GANs are more effective in data augmentation.

AEs and GANs are two typical structures of generative neural network models. They present a strong learning capacity in automatically capturing the stable and representative representations from cognitive biometric signals. These models are data-driven; therefore, the quality and generalization ability of the learned representations depend on whether the training samples are sufficient and diverse. GANs generate more clear and more realistic signal samples than those coming from AEs. Therefore, GANs are widely used for data augmentation, while AEs are usually used for feature extraction. Data augmentation aims to solve the problem of insufficient training samples in order to improve the classification performance of deep learning models. It is worth mentioning that data insufficiency is a practical problem and challenge in cognitive biometrics, as many databases have a very limited sample size due to difficulties in data collection. For example, for EEG data collection, subjects need to sit in a controlled environment wearing devices, which is a demanding process. Existing research shows that GANs are powerful tools for data augmentation for EEG, ECG, and PPG signals. Compared to AEs, GANs avoid the difficulty in loss function design, but the training of GANs is much more finicky and the data generation process cannot be directly controlled. Another advantage of AEs over GANs is that they offer a clear way to evaluate the quality of the model.

Compared to ICA, PCA, and GMMs, AEs and GANs are inferior in providing theoretical insights. However, the flip side of this drawback is the advantage of AEs and GANs in identifying valuable relationships that would be missed by statistical analysis or human preconceptions. In addition, developing ICA, PCA, and GMMs requires understanding the statistical characteristics of signals and checking the corresponding assumptions. AEs and GANs can bypass these steps and are able to obtain better representations from noisy data, for example, the ‘raw’ signals.

Table 4 summarizes all the handcrafted and automatic representations discussed in this section in terms of the methods, domains, major uses, and deep learning (DL) applicability. The domains describe what information is encoded and reflected in the representations and include the time, frequency, space, and hyper domains. Biosignals in cognitive biometrics are naturally observed in the time and space domains, as they are timeseries data collected from single or multiple physical locations. The frequency domain also plays a significant role, since these signals present important characteristics over specific frequency ranges. Furthermore, we use a hyper domain to denote latent information extracted through the multi-layer architecture of machine learning models or obtained after transformations and projections. The DL applicability indicates how applicable the representations are for use as input data for deep neural networks.

## 5. Pattern Recognition in Cognitive Biometrics

Recognition is another significant component in biometric recognition systems. This section reviews the methods for recognition of cognitive biometrics in diverse applications.

### 5.1. Conventional Methods

We summarize the following six types of conventional methods for cognitive biometric recognition. The representative studies are listed in Table 5.

#### 5.1.1. Similarity-Based Classifiers

Similarity-based classifiers predict the class label of a test sample based on its similarity to a set of labeled training samples and the pairwise similarity between the training samples. They are widely used for template (feature vector) matching in cognitive biometrics-based authentication. Different similarity measures and distance functions are applied, including cross-correlation [4,27,75], cosine similarity [24,48,53], Euclidean distance [61,72,82], Mahalanobis distance [22,60,72], and Manhattan distance [54,55,72]. The generalized similarity-based classifiers also include *k*-nearest neighbor (*k*NN) and dynamic time warping (DTW). *k*NN offers a better strategy, as it takes into account the most similar points to the input and applies a majority voting rule to fuse the decisions [46]. Moreover, to cope with the intra-person variations of features, improved *k*NN algorithms have been proposed, for example, fuzzy-rough nearest neighbor [149]. In these works, the matching templates (feature vectors) are composed of different handcrafted features. When cognitive biometric signals are directly used for classification without feature extraction, the DTW method is preferred. This is because DTW determines an optimal match between two signal timeseries, which can reduce the impact of signal misalignment and distortion on similarity measurements. Therefore, in cognitive biometrics, DTW is often used to classify EEG, ERP, and ECG signals instead of feature vectors [150]. Similarity-based methods are straightforward, fast to compute, and provide interpretable results. However, similarity based-methods are prone to over-fitting; hence, the model can be badly affected by outliers. In other words, their performance largely relies on the quality of the input signals or templates. These methods also suffer the curse of dimensionality.

#### 5.1.2. Discriminant Analysis (DA)

Discriminant analysis (DA) separates data samples of different classes by projecting data into a lower-dimensional space and maximizing the inter-class distance. It is popular in cognitive biometric recognition. Among the many linear and nonlinear discriminant functions, linear DA (LDA) is the most commonly used one and is recognized as a good choice for classifying handcrafted features in EEG-based person authentication and identification systems [84,86,95,105,151]. It is also used for classifying domain-specific and frequency-based features of ECG and PCG signals [35,36,152,153]. Quadratic DA (QDA) and its variants are also proposed for authentication [21]. LDA is also used for classifying domain-specific and frequency-based features of ECG and PCG signals [35,36,152,153]. DA methods are simple and fast; but require the normal distribution assumption for the input features.

#### 5.1.3. Support Vector Machines (SVMs)

Support vector machines (SVMs) project the data onto a space where the classes are separable using a hyperplane. The classification boundary is chosen to maximize the margin distance between the classes and the hyperplane. SVMs are even more popular than DA-based methods in cognitive biometric recognition. They have been used in a wide range of classification tasks for EEG [56,67,71,83], ECG [37,66,154], SCG [89] and EDA signals [43]. In these studies, the SVMs are equipped with linear kernels [48,83,155] and non-linear kernels including the radial basis function [48,67,69,71], sigmoid function, and polynomial function [48]. Linear SVMs are preferred in many studies, as they are computationally lightweight, while non-linear SVMs are able to fit a non-linear decision boundary between classes to solve more complex classification problems, although the computational cost is higher. The input of SVMs is various types of handcrafted features. SVMs usually work well with small datasets and when the input feature vectors have a clear margin of separation. However, performance will degrade severely when the input data has noise or overlaps between classes. In addition, the selection of a proper kernel can be computationally intensive.

#### 5.1.4. Neural Networks (NNs)

Neural networks (NNs) are computational algorithms that are characterized by a network structure composed of multiple layers of interconnected nodes, inspired by the human nervous system. They can model complex patterns from observational data through non-linear mapping of the input and output. They are gaining more and more attention in cognitive biometric recognition in recent years. Multilayer perceptrons (a feedforward structure) and Elman networks are used as classifiers in many cognitive biometrics studies based on EEG [62,87,90,98,156], ECG [157,158], EDA [43], and PPG signals [159]. Most of the NNs in these studies adopt the softmax function in the output layer and are trained under a cross-entropy regime. Some NNs use SVM and random forest in the output layer [156]. Other NNs used in cognitive biometrics include the linear vector quantizer [85]. Since many representations of cognitive biosignals are non-linear and contain complex dynamics, NNs are advantageous classifiers over similarity-based and DA-based methods. Disadvantages of NNs mainly lie in the computational complexity, difficulty in result interpretation, proneness to over-fitting, and the empirical nature of model development. Fortunately, researchers have started to tackle these problems. In Section 5.2, we will dig into deep learning algorithms, which are more powerful NN models with deeper and more complex architectures.

Other studies have applied the hidden Markov model (HMM), GMM, random forests (RFs), and other methods to cognitive biometric recognition based on ECG [38,158,160], PPG [161], SCG [89,162], PCG [79,163,164], EEG [52,83,165], and EDA signals [77]. Among them, HMMs, which are able to model raw sequential data and detect non-stationary changes, have been demonstrated to be efficient algorithms for classifying cognitive biological signals. There is usually a proper match between features and classifiers. We show the relationship between different handcrafted features and conventional recognition methods in Table 5.

**Table 5:** Summary of studies using handcraft representations with conventional recognition methods.

Representations (Handcrafted)		Recognition (Conventional Methods)				
		Similarity-Based	DA	SVMs	NN (Shallow)	GMM, HMM, RF, etc.
T	Signal	EEG [4,24,27]	EEG [105]	EEG [27]	EEG [156]	
	Domain-specific	ECG [35,158,166]		ECG [37,154]		ECG [38,158] PPG [161]
		PPG [167,168] PCG [39] EDA [43]	ECG [35,36,152]	EDA [43]	ECG [158] EDA [43]	SCG [162] PCG [163]
	Statistics	EEG [46]	EEG [44]	EEG [45,47,83]	EEG [59]	EEG [45]
		EDA [43]		EDA [43]	EDA [43] PPG [159]	
	AR	EEG [48,53,54,55,56]	EEG [21,48,49]	EEG [50,56]	EEG [51,56,98]	EEG [52]
	Entropy	EEG [60,61] EDA [43]		EEG [98] ECG [66] EDA [43]	EEG [59,62] EDA [43]	
F	PSD	EEG [48,53,61,72]	EEG [21,48,151]	EEG [48,67,69,71,169]	EEG [23,70,170]	EEG [45,165]
		EEG [56,68] EDA [43]	EEG [170,171]	EEG [45,50,56] EDA [43]	EEG [56,59] EDA [43]	
	HHT/EMD/DCT/MFC	EEG [55,56,92] PCG [39,101]	EEG [92]	EEG [47,56,155] ECG [160]	EEG [56,85,172]	ECG [157,160,173]
		ECG [35,36,157]	ECG [35]	ECG [157,174]	ECG [157]	PCG [79,164]
T + F	STFT/WT/WPD	SCG [89]	EEG [84,86]	EEG [83,175]	EEG [59,87,90]	EEG [83] ECG [78] PCG [79]
		ECG [38,82]	PCG [153]	EDA [77] SCG [89]		EDA [77] SCG [89]
T/F + S	Connectivity/graph	EEG [22,53,61,94,96]	EEG [21,95]	EEG [50,169]	EEG [98]	

T—time domain; F—frequency domain; S—space domain.

### 5.2. Deep Learning-Based Recognition

Table 6 summarizes the latest studies on deep learning-based recognition in cognitive biometrics, mainly covering methods based on deep feedforward neural networks (DFNNs), deep belief networks (DBNs), convolutional neural networks (CNNs), recurrent neural networks (RNNs), and graph convolutional neural networks (GCNNs).

#### 5.2.1. DFNN

Deep feedforward neural networks (DFNNs) are composed of an input layer, multiple hidden layers, and an output layer, wherein a node in a layer is connected to all nodes in the next layer and thus there are no loops in the network. They are simple and easy to implement, and are capable of capturing the complicated relationships of nodes between layers. DFNNs are commonly used in EEG and ECG cognitive recognition [98,157,176,177]. A DFNN-based method in [98] was used to extract EEG features from 1D input signals. Sun [176] developed a DFNN-based multitask learning method for EEG identification. Yang et al. [177] presented an EEG emotion recognition method based on DFNN. Pinto et al. [157] proposed a DFNN-based method for ECG identification and authentication.

#### 5.2.2. DBN

Deep belief networks (DBNs) are generative neural networks that are constructed by stacking RBMs, where each RBM block is aimed at learning the relationship between the input data and its output data. Compared with DFNN-based methods, DBNs are less popular in cognitive recognition [65,178]. Zheng et al. [65] proposed using a DBN-based method for EEG-based emotion recognition, which takes entropy features as input. Jindal et al. [178] developed a PPG-based identification method through a DBN. DBN-based cognitive recognition methods are mainly adopted in modeling 1D input signals or features.

#### 5.2.3. CNN

The key component in convolution neural networks (CNNs) is the convolution kernel/filter. Through sharing kernel weights, the size of the weights/parameters can be significantly reduced without losing network performance. Unlike DFNN and DBN, which are limited in their number of layers, it is easy to deploy more layers in a CNN network and achieve better performance. In addition, benefiting from the usage of convolution kernels, CNN networks can be used to effectively extract spatial relationship patterns/features from the input signals. CNNs have been used in various cognitive signal recognition such as EEG [17,98,179,180], ECG [88,181,182,183], PPG [184], SCG [80,89], and EDA [43,77]. The CNN-based methods proposed in [26,179,180,185] take as input 2D timeseries EEG signals to model spatial–temporal information. Wang et al. [98] developed EEG-based CNN networks by taking as input 2D univariate features and 2D connectivity matrices to model spatial information and connectivity information, respectively. In a hybrid method [17], a CNN network was utilized as a feature extractor to learn spatial information from the 3D wavelet scalogram series. Among CNN-based ECG recognition methods, da Silva Luz et al. [76] and Byeon et al. [88] utilized CNN networks to model temporal–spectral information for heart biometrics (1D timeseries heartbeat signal and 2D heartbeat spectrogram) and 2D CWT scalogram, respectively. The methods in [182,186] utilized CNN networks to model spatial-temporal information from 1D QRS timeseries ECG and 2D ECG data, respectively. Zhang et al. [181] developed a CNN network to extract spectral information from 1D DWT/AC features, while Sepahvand et al. [183] proposed an evolutionary CNN to model spatial–spectral information from 2D spectral connectivity of ECG signals. Everson et al. [184] proposed using a CNN network to preliminarily extract spatial information from 2D PPG signals. Maiorana et al. [80] and Hsu et al. [89] presented CNN networks for SCG-based recognition to learn temporal–spectral information from 2D STFT coefficients and 2D CWT coefficients, respectively. A CNN network was utilized for EDA-based recognition based on 1D handcrafted features [43], while Piciucco et al. [77] proposed a CNN-based recognition method by modeling temporal–spectral information from 2D STFT coefficients of EDA signals.

#### 5.2.4. RNN

Recurrent neural networks (RNNs) have been proposed to better handle time-series signals or sequential information, in comparison with DFNN, DBN, and CNN, which are focused on modeling relationships from a single-point input. The key concept behind RNNs is using state variables to model the relationship between past information and the current signals. One of the most successful RNN architectures is long short-term memory (LSTM), which contains three types of gates: input gates, forget gates, and output gates. These gates are used to preserve long-term information and forget short-term information. RNNs are mainly utilized to model the temporal relationship of cognitive signals [77,184,187,188,189]. Xing et al. [187] proposed an LSTM-based method for emotion recognition using multichannel EEG signals. Zhang et al. [188] proposed a spatial-temporal RNN method for emotion recognition by modeling the spatial and temporal dependencies of the input EEG signals. Hefron et al. [16] presented an LSTM-based method to improve cognitive workload estimation by considering the temporal information between 1D statistical features. Zhang et al. [17] combined an RNN with a CNN to model spatial–spectral-temporal information from 3D wavelet scalogram series of EEG signals. Salloum et al. [189] evaluated different RNN networks for ECG-based identification and authentication by exploiting spatial-temporal information for 2D timeseries ECG signals. Everson et al. [184] presented a PPG-based method using two LSTM layers to model the temporal relationships of PPG features. Furthermore, an LSTM-based network [77] was used to capture temporal-=spectral information from 2D STFT coefficients of EDA signals.

#### 5.2.5. GCNN

Graph convolutional neural networks (GCNNs) are an extension of CNNs and are designed to learn structural features from graphs. The graph is used to represent the input data in terms of nodes and edges. The nodes are aimed at encoding feature information, and the edges are encoded by an adjacency matrix to present the connections between nodes. Similar to the convolution in CNNs, special convolution filters are designed in GCNNs to learn features from neighboring nodes. Unlike conventional CNNs, which are designed for regularly structured data, GCNNs are proposed for irregular data where the number of nodes usually varies and the nodes are usually unordered. Wang et al. [98] proposed a GCNN-based EEG identification method to automatically capture deep intrinsic structural representations from EEG graphs. Experimental results show that the features extracted by the proposed GCNN are more robust than univariate features. Song et al. [190] presented a dynamical GCNN method for EEG emotion recognition by learning the intrinsic relationship between EEG channels. Zhong et al. [191] proposed a GCNN-based method for EEG emotion recognition, in which the GCNN is used to model the biological topology among different brain regions.

**Table 6:** Summary of deep learning-based representation learning and recognition in cognitive biometrics.

Signals	Input	Models	Encoded Information	Studies
EEG	1D CSP features	DFNN	Spatial	[176]
EEG	1D connectivity features	DFNN	Connectivity	[98]
EEG	1D entropy features	DFNN with subnetwork nodes	Spatial	[177]
EEG	1D entropy features	DBN	Spatial	[65]
EEG	2D timeseries	CNN	Spatial–temporal	[26,40,179,180,185,192]
EEG	2D timeseries	Conv.Enc. (adversarial learning)	Spatial–temporal	[171]
EEG	2D univariate features	CNN	Spatial	[98]
EEG	2D connectivity matrices	CNN	Connectivity	[98]
EEG	SAE latent representations	LSTM	Temporal	[187]
EEG	2D entropy features	RNN	Spatial–temporal	[188]
EEG	1D statistical features	LSTM	Temporal	[16]
EEG	3D wavelet scalogram series	3D-CNN+RNN	Spatial–spectral–temporal	[17]
EEG	graph representations	GCNN	Spatial–spectral/temporal	[98,190,191]
ECG	1D DCT-wavelet features	DFNN	Spectral	[157]
ECG	1D DWT/AC features	1D-CNN	Spectral	[181]
ECG	1D timeseries + 2D spectrogram	1D+2D-CNN	temporal–spectral	[76]
ECG	1D QRS timeseries	CNN	Spatial–temporal	[186]
ECG	2D embedding	CNN	Spatial–temporal	[182]
ECG	2D spectral connectivity	Evolutionary CNN	Spatial–spectral	[183]
ECG	2D CWT scalogram	Ensemble CNNs	Temporal–spectral	[88]
ECG	1D R peak timeseries	LSTM, RNN	Temporal	[189]
PPG	1D statistical features	DBN	Temporal	[178]
PPG	2D timeseries	CNN+LSTM	Spatial–temporal	[184]
SCG	2D STFT coefficients	CNN	Temporal–spectral	[80]
SCG	2D CWT coefficients	CNN	Temporal–spectral	[89]
EDA	1D handcrafted features	1D-CNN	-	[43]
EDA	2D STFT coefficients	CNN, CNN+LSTM	Temporal–spectral	[77]

### 5.3. Discussions

Compared to conventional recognition methods, DL-based methods offer advantages in the following three aspects. First, they can learn automatically from cognitive training data and rely less on expertise and knowledge about cognitive signals. Second, they can effectively model abstract representations/features for different tasks from massive cognitive training data. And finally, similar network architectures can be easily modified and used for different cognitive signals. The disadvantages of DL mainly lie in its dependence on a large training set, the difficulty in interpreting results, and high computational cost. The performance of DL-based recognition can be limited by an insufficient amount of training data, which is often a problem in cognitive biosignal datasets. Fortunately, existing results have shown that this problem can be solved by dataset augmentation using generative neural network models, especially GANs. In real applications, generalization is another issue, because DL-based methods tend to over-fit the training data and may not be able to generalize for new data collected in a different session or setup. Therefore, having diverse and sufficient training data is important.

More specifically, DFNN-based methods mainly focus on modeling 1D biosignals or features, as the size of parameters of DFNN is limited due to the full connection mechanism. Similar to DFNN-based methods, DBN-based cognitive recognition methods are mainly adopted for modeling 1D input signals or features. CNNs have proven flexible in cognitive biometrics recognition. First, they can be used in various cognitive signals, such as EEG, ECG, PPG, SCG, and EDA. Second, they can take diverse types of data as input, such as 1D/2D timeseries input, 2D univariate/bivariate features, and 3D input data. By combining with RNN or LSTM layers, CNN networks can be flexibly adapted to model multiple types of information simultaneously. LSTMs are mainly used to model the temporal relationships of time-series cognitive signals, especially 1D cognitive signals or features. Compared with the aforementioned DL-based methods, GCNN-based methods exploit the characteristics of the EEG signal graph. The experimental results obtained in [98,190,191] show that GCNNs can efficiently model the intrinsic relationships between different brain regions.

## 6. Open Research Directions

### 6.1. Deep Learning and Cognitive Biometrics

From the discussions in Section 4 and Section 5, it is observable that deep learning algorithms present the future trend of cognitive biometric recognition. We summarize the following research directions regarding deep learning for representation learning and recognition in cognitive biometrics.

- Dynamic representation learning and recognition. The existing feature extraction and classification methods are suitable for static mode, which assumes that the representations of the signals obtained in the test phase are subject to the same distribution as representations in the training phase. However, in practical applications, this assumption does not always hold because the cognitive biosignals can vary with the mental states of the user in the short term and be affected by aging in the long term. This will invalidate static features and hinder recognition performance. Dynamic representation learning and recognition which continuously incorporate new information from testing samples is a good way to maintain the stable performance of cognitive biometric recognition systems.
- Interpretations of the learned representations. Representations learned through deep learning models are generally complex since they incorporate hyper-domain abstractions of different levels in a hierarchical structure. In the image classification domain, algorithms such as layer-wise relevance propagation and weight visualization techniques are proposed to bring information about the fairness and interpretability of the models. This information is critical for researchers to gain useful insights into the model and understanding of the results and findings. Unfortunately, it is still an unsolved problem to interpret representations that are obtained by deep learning algorithms from cognitive biometric data. For example, emotions can be recognized by graph neural networks; however, it is unclear how to interpret the results and learned representations. The interpretability behind deep learning algorithms needs to be further studied so that the representations can be linked to existing domain knowledge. We argue that providing explanations of the discovered patterns from a neurobiological perspective is generally more important than just showing the classification/prediction accuracy.
- Multi-modality fusion through deep learning. Different biosignals may contain complementary information useful for a recognition task. In existing studies of multi-modal cognitive biometric systems, the fusion of different modalities is mainly performed at a decision level, where a majority voting strategy is used to determine a final decision based on decisions from individual modalities, or at the feature-level, where features extracted from individual modalities are concatenated into one vector for classification. Deep learning models offer a more flexible way to fuse information from different biosignals. Developing deep learning models, frameworks, and protocols to handle different biosignals at the same time is a new research area.
- Transfer learning in different applications and signals. The biosignals in cognitive biometrics have many common characteristics, so the deep neural network architectures being proposed are quite similar. As a result, one network model can be easily modified and used for a different signal or in a different application. It is a promising direction to design a unified deep learning model that can automatically fit various input cognitive signals. More importantly, deep learning points out a new research direction: transfer learning of cognitive biometrics, which concerns the storing and transferring of useful representations obtained during one recognition task to other tasks, and knowledge transfer from one signal to other signals. There are several studies that transfer deep learning models trained on the EEG-based identification task to authentication tasks by fine tuning the model for each subject. However, the range of possible applications of transfer learning is much greater than that.
- Federated learning for cognitive biometrics. The core idea of federated learning is to train machine learning models on separate datasets that are distributed across different devices or parties, which can preserve local data privacy to a certain extent [193]. Training deep learning models for cognitive biometrics usually requires massive data and computing resources. The transition from centralized training to distributed on-site learning will protect the privacy of each user while reducing the computing resources required by each device. Combining deep learning models with federated learning frameworks is a new direction in the field of cognitive biometrics.
- Deepfake algorithms: a booster or threat for cognitive biometrics? Deepfake algorithms refer to machine learning methods that are used to generate and manipulate fake human data, especially methods based on generative neural networks. On the one hand, existing research has demonstrated the potential of generative neural networks (such as GANs and AEs) in data augmentation, which is conducive to the training of DL-based recognition models, thereby improving the performance of cognitive biometrics. This is particularly important for cognitive biometrics due to the difficulty in collecting the biosignals. On the other hand, it is still an open research question whether the fake biosignals synthesized or generated by deepfake algorithms from other people’s data or publicly available datasets can spoof cognitive biometric systems. This could be a real concern, since the security of biometrics based on face and voice has been severely challenged by synthetic data generated through deepfake [194].

### 6.2. Security, Permanence, and Fusion of Cognitive Biometrics

Possible attacks on cognitive biometric systems include replay and spoofing attacks, jamming attacks, machine learning adversarial attacks, malware attacks, data injection attacks, and misleading stimuli attacks for systems involving sensory stimulation. Table 7 summarizes these attacks and where they occur in the recognition process. The security analysis of cognitive biometrics against these attacks is at the beginning stage, and it is worth continuing this line of research to propose countermeasures. In addition, designing privacy-preserving cancellable templates for cognitive biometrics is also an emerging topic.

Moreover, the permanence of cognitive biometrics is still an open research question. There are some preliminary findings regarding the permanence of biometrics based on EEG and ERP signals [24,27,53], but such studies are limited by the data availability. It is a major challenge to collect various cognitive biometric data from the same human subjects in different sessions across months and even years. Open data sharing platforms such as the PhysioNet offer potential in allowing large-scale evaluation of cognitive biometrics and permanence analysis.

Finally, the fusion of multiple cognitive biometrics is a promising direction. Multi-modality fusion improves recognition accuracy and robustness, and also provides a potential countermeasure for presentation attacks. Furthermore, since cognitive biometrics present an inherent liveness detection function, it is useful to design proper protocols and frameworks to integrate cognitive biometrics in traditional biometric systems as a liveness detection module.

## 7. Conclusions

Cognitive biometrics is a new branch of biometric technology and there is a great need to review the latest developments in this field. In this article, we presented a comprehensive review of cognitive biometrics, covering all the major biosignal modalities and applications. A taxonomy was designed to structure the corresponding knowledge and guide the survey from signal acquisition and pre-processing to representation learning and pattern recognition. First, we defined the scope of cognitive biometrics and summarized the biosignals considering their origin, sensing technologies, sensing locations, physical signals, and elicitation/acquisition protocols. The review breaks the barriers between different fields and reveals the relationship between different biosignals. Second, due to the inherent cognitive and emotional information carried by cognitive biometric data, our discussion on cognitive biometrics was not limited to authentication and identification, but also included a broader range of applications in human–computer interaction, adaptive control, and decision and health aid. We summarized the functions of cognitive biometrics module in these application scenarios and linked them to the corresponding recognition tasks. The extension of the above two aspects allowed us to restructure the knowledge regarding representation extraction/learning and recognition in cognitive biometric recognition across domains, biosignals, and applications. A systematic review of representation extraction/learning and recognition methods was then carried out. We provided a unified view of the methodological advances across various biosignals and applications, facilitating interdisciplinary research and knowledge transfer across fields. Particularly, we investigated recent works on generative models and various deep learning models, and discussed how they are used in cognitive biometric recognition. Finally, we discussed future research directions in cognitive biometrics in two aspects: the deep learning-related directions and other issues regarding the security, fusion, and permanence of cognitive biometrics.

## Figures and Tables

**Table 1:** Biosignals for cognitive biometrics.

Sensing Technique	Origin	Sensing Location	Physical Signal	Elicitation Protocol
EEG	Brain	Scalp	Electrical	Resting/Internal/External
ECG	Heart	Chest	Electrical	Resting
PPG	Heart	Finger	Optical	Resting
PCG	Heart	Chest	Acoustic	Resting
SCG	Heart	Chest	Mechanical	Resting
EMG	Muscle	Arm	Electrical	Resting
EDA	Skin	Fingertip	Electrical	Resting/External
EOG	Eye	Around eyes	Electrical	Internal/External

Resting: spontaneous activity. Internal stimulation: using internal or volitional tasks to elicit particular responses. External stimulation: using external sensory stimuli to elicit particular responses.

**Table 2:** Public databases for cognitive biometrics.

Database	Signal (#Ch.)	Device/Sensor	Sampling rt.	Protocol/Condition	#Subj.	#Sess.	Year
SEED-IV	EEG (62)	ESI NeuroScan ${}^{†}$	200 Hz	Movie video	15	3	2013
BED	EEG (14)	Emotiv EPOC+ ${}^{‡}$	256 Hz	Resting, affective stimuli, mathematical computation, visual stimuli	21	3	2021
BCI2008 GrazA	EEG (22), EOG (3)	Unclear	250 Hz	Resting, motor imagery	9	2	2008
BCI2008 GrazB	EEG (3), EOG(3)	Unclear	250 Hz	Resting, motor imagery	9	5	2008
MMIDB	EEG (64)	BCI2000 ${}^{‡}$	160 Hz	Resting, motor imagery	109	1	2009
Alcoholism	EEG (64)	Unclear	256 Hz	Picture stimuli	122	1	1999
DEAP	EEG (32), EOG (4), EMG (4), EDA (1)	Biosemi ActiveTwo ${}^{†}$	512 Hz	Music video	32	1	2012
Keirn and Aunon	EEG (6), EOG (1)	Unclear	250 Hz	Resting, problem solving, geometric figure rotation, visual counting, mental letter composing	7	1	1989
BCI CSU	EEG (32), EOG (4)	Biosemi ActiveTwo ${}^{†}$	1024 Hz	Resting, P300, letter counting	9	1	2012
	EEG (8)	g.Tec g.GAMMAsys ${}^{†}$	256 Hz				
	EEG (19)	Neuropulse Mindset ${}^{‡}$	512 Hz				
MAHNOB-HCI	EEG (32), ECG, EDA	Biosemi ActiveTwo ${}^{†}$	1024 Hz	Movie video	27	1	2012
DREAMER	EEG (14) ECG (4)	Emotiv EPOC+ ${}^{‡}$ Shimmer ${}^{‡}$	128 256 Hz	Movie video	23	1	2018
European ST-T	ECG (2)	Unclear	250 Hz	Ambulatory ECG	79	1	2009
MIT-BIH	ECG (2)	Unclear	360 Hz	Ambulatory ECG	47	1	2005
ECG-ID	ECG (1)	Unclear	500 Hz	Resting	90	1–20	2014
CYBHi	ECG (2) EDA	Dry electrode ${}^{‡}$ Ag/AgCl ${}^{‡}$	1 kHz	Undisclosed	125+	2	2014
UofTBD	ECG (1)	Vernier ECG sensor ${}^{‡}$	200 Hz	Postures and motions	100	1–6	Unclear
PTB	ECG (14)	Wet electrode ${}^{‡}$	1 kHz	Clinical condition	290 (52 healthy)	1	2004
AHA	ECG (2)	Wet electrode ${}^{‡}$	250 Hz	Ambulatory ECG	155	1	2003
DRIVEDB	ECG (1), EMG (1), EDA (1)	Wet electrode ${}^{‡}$	496 Hz	Driving condition	17	1	2008
BioSec. PPG	PPG (1)	Plux pulse sensor ${}^{‡}$	n.a.	Office environment	100, 170	2	2020

^†^ medical-grade devices. ^‡^ consumer-grade devices.

**Table 3:** Connectivity metrics.

Connectivity Metrics	Perspectives	Domains	Value Ranges	Study
Pearson’s correlation	Linear correlation	Time	[−1,1]	[21,93,96,98]
Granger causality	Causal relationship	Time	[0,∞)	[25]
Mutual information	Information theory	Time	[0,∞)	[21]
Spectral coherence	Coherence between spectral components	Frequency	[−1,1]	[22]
Phase locking value	Variability of relative phase	Phase	[0,1]	[93,95,96,97,98]
Phase lag index	Interdependence of relative phase	Phase	[0,1]	[93,94,96]
Phase synchronization index	Deviation of relative phase	Phase	[0,1]	[96,100]

**Table 4:** Summary of representation extraction and learning methods discussed in this section.

Representation	Foundations	Domains				Major Use	DL Applicability
		Time	Frequency	Space	Hyper		
Domain-specific	Handcrafted	✓				Extraction	Low
Descriptive statistics	Handcrafted	✓				Extraction	Low
AR models	Handcrafted	✓				Extraction	Median
Entropy	Handcrafted	✓				Extraction	Low
PSD, FFT	Handcrafted		✓			Extraction	Median
EMD, HHT	Handcrafted		✓			Extraction	Median
DCT, MFC	Handcrafted		✓			Extraction	Median
STFT (spectrogram)	Handcrafted	✓	✓			Extraction	High
WT (scalogram), WPD	Handcrafted	✓	✓			Extraction	High
Connectivity, graph	Handcrafted	✓	✓	✓		Extraction	High
LDA	Auto. (supervised)				✓	transform	Low
CSP	Auto. (supervised)			✓	✓	Extraction	Median
NN	Auto. (supervised)				✓	Extraction and classification	High (integrated)
PCA, ICA	Auto. (unsupervised)				✓	Transform, preprocessing	Low
Clustering	Auto. (unsupervised)				✓	Pre-classification, wave detection	Low
RBNs	Auto. (unsupervised)				✓	Extraction	High (integrated)
AEs	Auto. (unsupervised)				✓	Extraction, data augmentation	High
GANs	Auto. (unsupervised)				✓	Data augmentation	High

**Table 7:** Attacks on cognitive biometric systems.

Attacks	Definitions	Stage
Replay attack	Reuse victim’s biometric template collected previously to impersonate the victim	Acquisition
Spoofing attack	A presentation attack that uses fake data to impersonate the victim	Acquisition
Jamming attack	Override the legitimate signals emitted from electrodes with false data	Communication
Misleading stimuli attack	Present malicious sensory stimuli to users to elicit specific responses	Acquisition
Adversarial attack	Manipulate machine learning systems by crafted inputs to disrupt their normal functioning	Recognition
Signal injection attack	Inject false data into the biometric system to alter its behavior and output	Recognition
Malware attack	Use hardware/software/firmware to gain access to devices to perform malicious actions	System

## Data Availability

Not applicable.
